# Quantifying variation of non-point source pollution and its impact factors: A study of Nansi Lake Basin

**DOI:** 10.1371/journal.pone.0318691

**Published:** 2025-02-25

**Authors:** Jiachen Liu, Yuan Tian, Rongqiang Ma, Wenhui Xie, Dongchao Wang, Luoan Yang, Xinyu Wang, Le Yin, Baolei Zhang

**Affiliations:** 1 College of Geography and Environment, Shandong Normal University, Jinan, China; 2 Shandong Provincial Territorial Spatial Ecological Restoration Center, Jinan, China; University of Peshawar National Centre of Excellence in Geology, PAKISTAN

## Abstract

Agricultural non-point source (NPS) pollution directly affects the quality of soil and water, ecological balance and human health, and is a key challenge to achieve sustainable environmental development and efficient resource management. Taking the Nansi Lake Basin (NLB) as the study area, this study explores the main sources of agricultural NPS pollution and its influencing factors, aiming to provide scientific basis for the management of water resources in the basin. Current studies usually use the runoff pollution partitioning method to estimate agricultural NPS pollution loads in runoff, but the accuracy of the analyses is limited by the incompleteness of water quality monitoring data, especially the lack of complete runoff records in some years. To compensate for this deficiency, this study simulated the river runoff based on the Long-Term Hydrological Impact Assessment (L-THIA) model, and applied the simulation results to the quantitative calculation of agricultural NPS pollution loads after verifying the model reliability through accuracy calibration. Based on L-THIA model, the spatial and temporal distribution data of agricultural NPS pollution in the basin from 2010 to 2020 were obtained, the distribution characteristics of chemical oxygen demand (COD) and ammonia nitrogen (NH_3_-N) were quantitatively assessed, and the impacts of natural and socio-economic factors on them were analyzed. A regression model was developed to simulate future agricultural NPS pollution through multiple regression analysis. The results showed that the total agricultural NPS pollution in the NLB showed an increasing trend during the study period. In particular, among the socio-economic factors, COD and NH_3_-N were significantly correlated with fertilizer application, pesticide use, rural employment and total population. Among the natural factors, topographic index, watershed area and gully density were positively correlated with pollutants, while slope and soil organic matter were negatively correlated. The results of this study raise awareness of the contribution of influencing factors and allow researchers and planners to focus on the most important NPS pollution sources and influencing factors. The study provides an important reference for the prevention and control of agricultural NPS pollution in the NLB, which is of great practical importance.

## 1. Introduction

Non-point source (NPS) pollution is one of the major sources of pollution in the aquatic environment worldwide, especially in regions with intensive agricultural production activities, where its impact on the quality of water bodies and soils becomes more and more significant [1]. As a major source of NPS inputs, agricultural activities release large amounts of nitrogen and phosphorus into aquatic environments, leading to various problems such as toxic algal blooms, oxygen depletion [2] and loss of biodiversity [3]. It is estimated that 30%–50% of the world’s land is affected by NPS [4], and about 60% of water body impairments are attributed to NPS pollution [5]. In Denmark, 94% of the nitrogen loads and 52% of the phosphorus loads from 270 rivers are due to NPS processes [6]. Similar trends were observed in the Netherlands, where NPS pollution accounted for 60% of total nitrogen pollutants and 40–50% of total phosphorus pollutants [7]. In China, NPS pollution is estimated to account for 81% of ammonia nitrogen (NH_3_-N) and 93% of phosphorus in total water pollution [8]. More than 60% of lakes are affected by eutrophication, half of which is caused by NPS pollution [9]. Therefore, NPS pollution is a growing global concern [8,10].

In recent years, significant progress has been made in the study of agricultural NPS pollution, which is mainly reflected in the continuous optimization of pollution load estimation methods and the wide application of modelling tools [11]. NPS pollution models can simulate the migration process of NPS pollution, predict the impact of pollutant loads on water quality, and provide effective means for the prevention and management of NPS pollution [12]. At present, the construction of NPS pollution model, NPS pollution spatial and temporal distribution simulation, impact assessment and pollution management, so as to promote the control and management of NPS pollution, is the focus of NPS pollution research [13]. With the development and application of geographic information system (GIS) technology, many hydrological and water quality models have been developed and integrated with GIS, such as the Storm Water Management Model (SWMM) [14], Hydrological Simulation Program-FORTRAN (HSPF) [15], Agricultural non-point Source (AGNPS) [16], and Soil and Water Assessment Tool (SWAT) [17]. These models bring great convenience and high evaluation accuracy to the simulation of spatial and temporal distribution of NPS sources, and provide a feasible method for the prevention and control of NPS pollution in watersheds [18]. However, these models require high input data, not only high-resolution land use, soil and topographic data, but also a large amount of detailed pollution data, meteorological data and agricultural practice data [19]. The difficulty of data collection limits the application of these models to a certain extent, and may even lead to significant deviations in simulation results if the data are incomplete or the model assumptions are consistent with the actual situation [20]. Therefore, there is an urgent need for methods to extract NPS pollution data from existing water quality monitoring data. Long-Term Hydrological Impact Assessment (L-THIA) models can be constructed using land use, soil type, and precipitation data, and their outputs include runoff volumes and NPS pollution loads. Despite the relative simplicity of its mechanism, the model can accurately assess long-term average surface runoff at the basin or urban scale. The L-THIA model was originally developed by Harbor [21] to simulate the impacts of land use changes on groundwater and wetland runoff. With the advancement of technology, the L-THIA model has been integrated with GIS, enabling the GIS-based L-THIA model to first estimate the runoff from smaller watersheds in the study area and then calculate the total runoff for the whole area, thus obtaining more realistic and reliable simulation results [22]. Many researchers have introduced the L-THIA model to simulate pollution in Chinese basins such as Miyun Reservoir area [23], Tao’er River Basin [24], Qingdao City [25], Guanlan River Basin [26], and Shiqiao River Basin [27], and achieved better results.

In addition, existing studies have gradually analyzed the influencing factors of agricultural NPS pollution. NPS pollution is characterized by stochasticity, universality and uncertainty [28], which leads to the complexity and diversity of its influencing factors. Many studies only focus on single or small number of pollutants such as nitrogen and phosphorus [29], ignoring the interaction between multiple pollutants and their comprehensive impact on the ecology of water bodies. Therefore, studying the impacts of NPS pollution involves many aspects, including its generation, transport and transformation mechanisms [30]. Identifying NPS pollution, especially understanding its development trends and determining its influencing factors, is a fundamental scientific challenge that is essential for solving China’s deteriorating water environment problems. Unlike point source pollution, which can be traced back to a single source, such as discharges from wastewater treatment plants [21], NPS pollution originates from multiple sources and is influenced by a range of natural environmental and socio-economic factors. There are many studies on the identification of factors affecting agricultural NPS pollution [27,31–33]. For example, some scholars have attempted to analyze how farmers’ behavior affects agricultural nitrogen and phosphorus pollution [34,35], while others have used the environmental Lorenz curve to assess the relationship between environmental quality and economic growth [22]. However, this part of the research only cuts from a single dimension and lacks a systematic exploration of the complex interactions between socio-economic factors and natural environmental factors, while ignoring the combined effects of watershed characteristics and socio-economic factors.

Nansi Lake is the largest freshwater lake in northern China and serves as a key hub along the eastern route of the South-North Water Transfer Project. As such, the water quality in the Nansi Lake Basin (NLB) is critical to the health of populations in many cities along the project route and directly influences the success of the overall water transfer initiative. Consequently, many scholars are focused on addressing water pollution prevention and control in the NLB. Based on water quality monitoring data, typical basin characteristics, and socio-economic data from the NLB, this study aims to (1) differentiate between NPS pollution and point source pollution using hydrological and water quality data, (2) quantitatively analyze the changes in chemical oxygen demand (COD) and NH_3_-N NPS pollution in Nansi Lake, and (3) identify the key factors influencing COD and NH_3_-N NPS pollution. This study will not only provide a scientific foundation for controlling water pollution in Nansi Lake but also help policymakers and researchers better understand the causes of water quality deterioration, particularly the increasing impact of NPS pollution.

## 2. Materials and methods

### 2.1. Study area

Nansi Lake (34°27~35°20’N, 116°34~117°21’E) is the largest freshwater lake in Shandong Province, and even in North China, and one of the ten largest freshwater lakes in China, with a maximum water area of about 1266 km^2^, accounting for 45 per cent of Shandong Province’s freshwater area. The NLB covers four cities, Jining, Heze, Zaozhuang and Tai’an, with an area of about 29000 km^2^, of which Tai’an contains only Ningyang County. The study area has a temperate monsoon climate, with average annual rainfall ranging from 590–820 mm. The main land use types are cropland and construction land (the secondary land use classification system is from the Chinese Academy of Sciences), and as of 2020, the total population is about 22.21 million people, of which the agricultural population accounts for 76.03 per cent of the total population.

### 2.2. Datasets

The land use data of Beijing came from Wuhan University (https://zenodo.org/records/12779975) and the land use types included 9 categories (cropland, forest, shrub, grassland, wetland, water, impervious, barren, snow/ice). We used the ArcGIS reclassification function to reclassify the land use data, and used the random point selection function to test the accuracy, and the accuracy of the interpretation results was more than 90.8%. Rainfall data were obtained from the Shandong Meteorological Data Monitoring Centre. Hydrological monitoring data were obtained from the Shandong Environmental Monitoring Centre, including water quantity and water flow rate at key cross sections of each river. 2010–2020 data on fertilizer application, poultry farming, investment in environmental protection, value added of the primary industry, value added of the secondary industry, and agricultural population were obtained from the statistical yearbooks of each prefecture-level city. Water quality monitoring data were obtained from the Shandong Environmental Monitoring Center, and the water quality factors include COD and NH_3_-N. Rainfall data, hydrological monitoring data, water quality monitoring data, and statistical yearbook data were processed using Excel to remove outliers and then used directly.

### 2.3. Methods

#### 2.3.1. L-THIA model.

In order to compensate for the missing runoff volume in the water quality monitoring data, this study simulated the runoff volume of the river based on the L-THIA model, and used it for the calculation of the runoff volume of the agricultural NPS pollution after the accuracy check (Table 1). The L-THIA model was designed by Harbor [20] based on the SCS-CN (Curve Number) model, which is the key parameter of the SCS model, and its value quantifies the subsurface conditions of the basin, and reflects the impact of subsurface conditions on streamflow production and catchment with a quantitative indicator. The L-THIA model is based on the ratio of the actual infiltration volume (F) to the maximum possible soil infiltration volume (S), which is equal to the ratio of the actual surface runoff depth (Q) to the maximum possible runoff depth [36]:

**Table 1:** Meaning of each parameter of the L-THIA model.

Name	Equation	Meaning
Precipitation (P)	$P=I_{a}+F+Q$	$I_{a}$ is the initial rainfall loss (mm), *F* is the actual infiltration (mm).
Antecedent soil moisture conditions (AMC)	$AMC=\overset{5}{\underset{i=1}{\sum }}P_{i}$	Pre-soil moisture was judged based on the rainfall in the previous 5 days, and the pre-rainfall index AMC was introduced, with $P_{i}$ being the amount of rainfall (mm) in the last 5 days.
	$AMCI:CN_{1}=\frac{4.2*CN}{10-0.058*CN}$ $AMCIII:CN_{3}=\frac{23*CN}{10+0.13*CN}$	Changes in CN values are largely influenced by the AMC of soil pre-water conditions, assuming that the AMC values are related to the first five days of rainfall, and dividing the AMC into three classes according to the rainfall situation in the five days before the sub-flood $CN_{1}$ -soil drought but greater than the wilting point situation; $CN_{2}$ -soil moderately wet situation; $CN_{3}$ -soil wet situation with soil saturated with osmotic saturation. In this study, we extracted the first 5 days’ rainfall of each land use type from the data of rainfall monitoring stations, and revised the built-in parameter CN of the model with reference [37] to the value of runoff volume of NLB in the study of water resources characteristics of NLB, and the CN values of the basin were taken as shown in Table 2.
Surface runoff depth (Q)	$Q=\frac{\left(P-I_{a}\right)²}{P-I_{a}+S}$	*S* is the maximum possible soil infiltration (mm).


$$\frac{F}{S}=\frac{Q}{P-I_{a}}$$ (1)

The calculation of each parameter is shown in the Table 1 below:

**Table 2:** CN values taken in the NLB.

Land use	Soil wetness		
	Dry	Medium wet	Wet
Cropland	64	75	82
Forest	57	73	82
Grassland	68	79	86
Townland	77	85	90
Rural neighborhoods	46	65	77
Transport land	76	85	89
Water	98	98	98
Unused land	77	86	91

#### 2.3.2. NPS pollution estimation.

In this study, based on the mass balance of pollutants, the amount of agricultural NPS pollution in runoff was obtained using the runoff pollution partitioning method, which identifies the pollution load in the river monitoring section as consisting of point source and NPS pollutants, since the number of pollutants discharged into the river from point source pollution is relatively stable and fixed [20]. Agricultural NPS pollution mainly occurs during periods of abundant water level. During periods of low water level, the average concentration of agricultural NPS pollutants is negligible, and the monitoring values are used only to indicate the concentration of point source pollutants. Based on this assumption, the separation process is described in equations (2) to (5).

The total amount of pollutants in the runoff can be calculated as:


$$L=\overset{12}{\underset{i=1}{\sum }}C_{i}\times Q_{i}$$ (2)

where *L* is the total amount of pollutants in runoff (kg), $C_{i}$ is the monthly monitoring concentration (g/L); $Q_{i}$ is the monthly runoff volume (m^3^); and *i* denotes the month.

The amount of point source pollution can be calculated as:


$$PS=\overset{4}{\underset{j}{\sum }}C_{j}\times Q_{j}$$ (3)

where PS is the point source pollution load (kg); $C_{j}$ the concentration of the pollutant in the dry season (g/L), $Q_{j}$ is the dry season runoff volume (m^3^), and *j* is the dry season, which in this study is February to May.


NPS=L−PS (4)

Therefore, the amount (kg) of agricultural NPS pollutants can be calculated as follows:


$$L_{NPS}=\frac{\sum_{i=1}^{n}UNPS_{i}}{A_{i}}\times A$$ (5)

The total amount of agricultural NPS pollutants in the NLB was calculated as follows:

$L_{NPS}$ is the total amount of specific NPS pollutants in the entire NLB; *i* is an area of a control unit; $UNPS_{i}$ is the amount of specific NPS pollutants in subbasin *i*; *A* is the NLB; and *n* is the number of subbasin control units.

#### 2.3.3. Selection of influencing factors.

We selected 26 factors as characteristic factors that may affect the basin through expert consultation and based on the literature [38] and previous studies, and then performed the Breusch-Godfrey LM test [39] to determine the autocorrelation between the analyzed factors. Finally, we used a method employing the Pearson correlation test to select basin characteristics that were statistically significant for NPS pollutants. The factors are shown in the Table 3 below:

**Table 3:** Influence of characteristic factors on agricultural NPS pollution.

Type	Code	Short name	Name
Socio-economic factors	X1	YTP	Total population at the end of the year
	X2	PD	Population density
	X3	GDP	Gross Domestic Product
	X4	PGDP	Per capita GDP
	X5	PPR	Proportion of primary sector
	X6	PSR	Proportion of secondary sector
	X7	PTR	Proportion of tertiary sector
	X8	RPA	Rural population
	X9	NAPR	Proportion of non-agricultural population
	X10	YAP	Rural practitioners
	X11	FAA	Total fertilizer use
	X12	PAA	Total pesticide use
	X13	RECA	Total rural electricity use
Natural factors	X14	BD	Patch density
	X15	LSI	Landscape shape index
	X16	MPS	Mean plaque size
	X17	SLOPE	Slope
	X18	PRE	Precipitation
	X19	NDVI	Normalized Difference Vegetation Index
	X20	DA	Basin area
	X21	TD	Trench density
	X22	SOMC	Soil organic matter
	X23	PAL	Proportion of cropland
	X24	PGAL	Proportion of garden land
	X25	PFL	Proportion of forest land
	X26	PGRL	Proportion of grassland

#### 2.3.4. Multiple regression analysis and testing.

Based on the physical and socio-economic characteristics of the study area, COD and NH_3_-N pollution in 11 control units was investigated using multiple regression analysis. In general, generalization between NPS pollution and basin characteristics may yield a population-like model for the dataset rather than a single best model for stepwise regression analysis. After autocorrelation and correlation tests, several multiple linear regression equations were developed in the software SPSS and expressed as:


$$L_{a}=a_{1}X_{1}+a_{2}X_{2}+a_{3}X_{3}+\ldots +a_{n}X_{n}+C$$ (6)

where La is the number of COD and NH_3_-N NPS pollution (kg), $a_{1}$, $a_{2}$ … $a_{n}$ are regression coefficients, $X_{1}$, $X_{2}$ … $X_{n}$ are basin characteristics and *C* is a constant term. The data were analysed as multiple linear regression by applying a logarithmic transformation to all variables to satisfy the normality requirement and establishing a regression equation of the form:


$$\log\left(L_{b}\right)=\log\left(C\right)+b_{1}\log\left(X_{1}\right)+b_{2}\log\left(X_{2}\right)+b_{3}\log\left(X_{3}\right)+\ldots ++b_{n}\log\left(X_{n}\right)$$ (7)

We used R^2^ checks of these equations to select models with better-fitting simple independent variables. Finally, as a result of the analyses, the equations for each pollutant were retained and the significance of the individual p-values for the remaining variables was tested.

The predictive ability of the models was assessed using the relative error (RE) and the Nash efficiency coefficient [40], calculated as:


$$RE=\frac{L_{s}-L_{0}}{L_{0}}\times 100$$ (8)

$$E_{NS}=1-\frac{\sum_{i=1}^{n}\left(L_{01}-\bar{L_{s1}}\right)²}{\sum_{i=1}^{n}\left(L_{01}-\bar{L_{0}}\right)²}$$ (9)

where $L_{01}$ is the NPS pollution monitoring value, $L_{s1}$ NPS pollution modelling value, control unit NPS pollution average monitoring value, *n* is the total number of years.

### 2.4. Data preprocessing

In this study, the basin analysis tool was used to divide sub-basins by using the river water quality monitoring points in the NLB as outlets, combined with the methods of customizing the major rivers in the plains and determining the catchment basins. Since part of the NLB is dominated by plain terrain, the sub-basin delineation method based on basin analysis tool is not effective. Therefore, we referred to related studies [32] and conducted sub-basin delineation based on the basin control cross sections in Shandong Province delineated by Shandong Provincial Environmental Protection Department, and corrected the results of the sub-basin delineation of the Nansi Lake sub-basin considering the availability of the data to finally delineate 11 sub-basins.

## 3. Results

### 3.1. Average total pollution from NPS in the NLB

The average pollution amount of NPS of COD in the basin of the Nansi Lake from 2010 to 2020 is shown in Table 4. The average pollution amount of NPS of COD in the central part of the basin is relatively good. Overall, the NPS pollution in the central part of the basin is relatively good, and the spatial distribution of pollution is generally characterized as ‘low in the center, high on both sides, and more concentrated’. In 2010, the three sub-basins with the largest amount of COD NPS pollution were Dongyu River, Wanfu River and Guangfu River. The three sub-basins with the smallest amount of NPS pollution were Chengguo River, Beisha River, and Shushui River Basin. In 2020, the largest amount of NPS pollution of COD in the NLB will continue to be the Dongyu River, followed by the Zhuzhaoxin River Basin, which has the largest number of COD. In 2020, the largest amount of COD NPS pollution in the NLB is still the Dongyu River, followed by the Zhuzhaoxin River and Xinxue River, showing a substantial increase in the amount of pollution relative to that in 2010, and the smallest amount of COD NPS pollution in 2020 will be in the basins of the Zhushui River, the Baima River, and the Beisha River, and the size of the amount of pollution did not fluctuate too much in comparison with that in 2010. The percentage of COD NPS pollution is maintained at 73.09%~83.48%.

**Table 4:** The average amount of COD and NH_3_-N pollution from 2010 to 2020.

Basin control unit	COD pollution/t/m³	NPS COD pollution		NH_3_-N pollution/t/m³	NPS NH_3_-N pollution	
		Amount/t	Proportion/%		Amount/t	Proportion/%
Baima River	18240.72	14675.32	80.45	44.85	34.94	77.90
Beisha River	20540.16	16144.59	78.60	115.21	68.61	59.55
Chengguo River	20491.29	15373.49	75.02	54.32	39.16	72.09
Dongyu River	188737.27	157556.96	83.48	193.89	135.19	69.73
Guangfu River	43519.39	35739.08	82.12	108.15	83.82	77.51
Liangji River	52004.53	40730.48	78.32	83.67	64.59	77.20
Si River	52443.83	41599.53	79.32	65.64	48.18	73.41
Wanfu River	80628.45	64251.08	79.69	164.75	129.12	78.37
Xinxue River	84368.17	64232.00	76.13	332.13	245.61	73.95
Zhushui River	16267.87	13361.36	82.13	69.16	45.32	65.52
Zhuzhaoxin River	158278.12	115686.72	73.09	236.49	139.74	59.09
Total	735519.79	579350.62	78.77	1468.26	1034.28	70.44

The time series changes of NH_3_-N NPS pollution in the basin of the Nansi Lake from 2010 to 2020 are shown in Table 4. The overall distribution of NH_3_-N pollution in the central and eastern parts of the basin is generally in good condition. Overall, the NH_3_-N pollution in the central and eastern part of the basin is generally in good condition, and the overall distribution of pollution is characterized by ‘high in the west, low in the center, locally concentrated and unevenly distributed’. The most obvious change in the amount of NH_3_-N pollution from 2010 to 2020 was in Xinxue River, where the amount of NH_3_-N pollution showed an upward trend, increasing by about 13 times. The three sub-basins with the largest amount of pollution are Xinxue River, Chenggu River, Baima River and Si River Basin in the order of the largest amount of pollution, the amount of pollution is less than 100 t, and the proportion of NH_3_-N pollution from unorganised discharge stays in the range of 59.09% to 78.37%.

### 3.2. Temporal changes in total NPS pollution in the NLB

**Fig 1:**
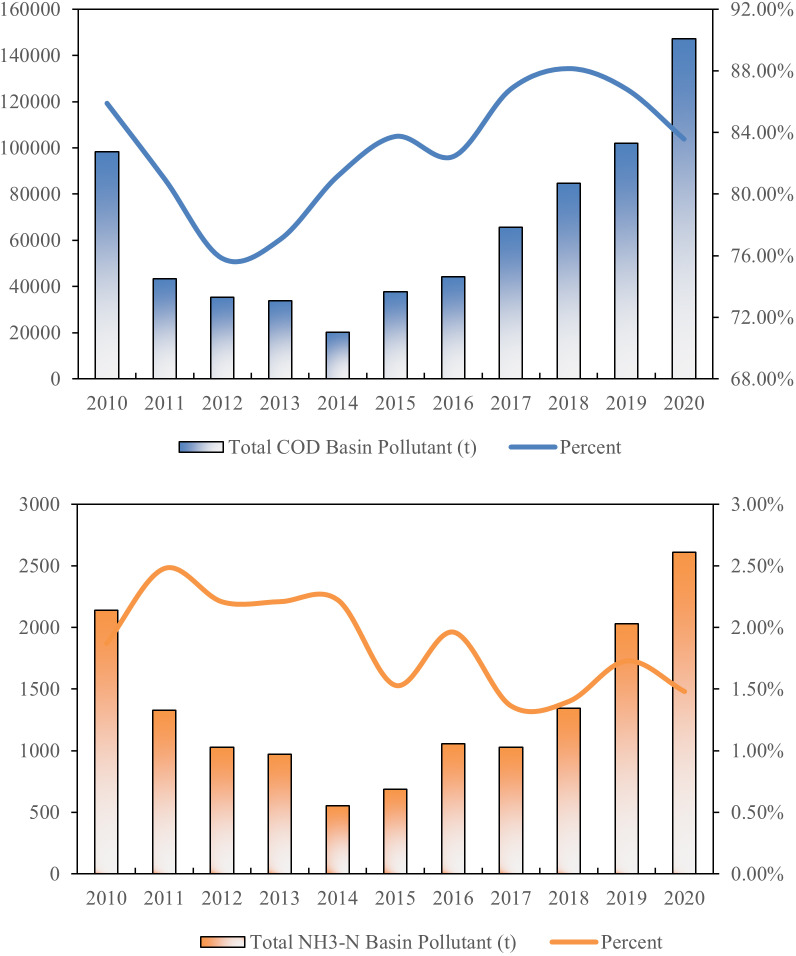
Temporal changes in total runoff pollution in NLB, 2010–2020.

By analyzing the statistical data of the NLB as well as the monitoring data of the river, this study reveals the spatial and temporal evolutionary characteristics of the pollutants in the basin, which provides a key basis for further analyses. The time span of the study is 2010–2020, and the time series changes of total COD and NH_3_-N runoff pollutants within the NLB are shown in Fig 1 (Temporal changes in total runoff pollution in NLB, 2010–2020). In terms of the overall trend, the changes in the values of these two indicators generally show an upward trend. Specifically, the values of each pollutant change along a trajectory of first decreasing and then increasing, and show significant differences between the magnitude of increase and decrease. Our study found that COD is the main pollutant in the basin. In 2020, the fluctuation of COD emissions was more significant. Compared to COD, the change in NH_3_-N emissions was relatively small, with an overall rate of change of 92%.

**Fig 2:**
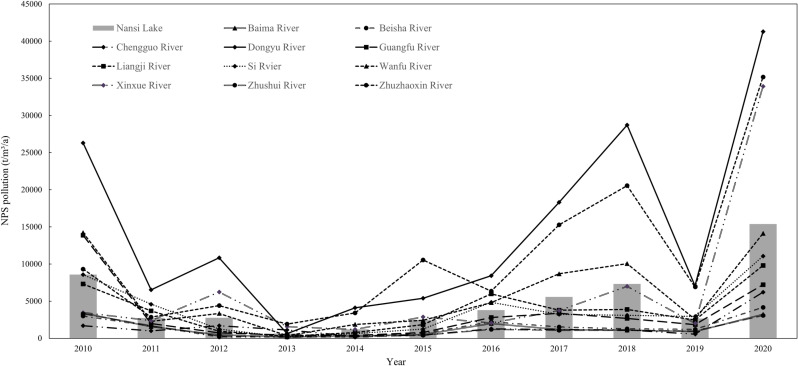
Temporal changes in total runoff COD pollution in each sub-basin, 2010–2020.

According to the time-series change process of COD pollution volume in the NLB from 2010 to 2020 (Fig 2 Temporal changes in total runoff COD pollution in each sub-basin, 2010–2020), the COD pollutant emissions in the central and eastern parts of the basin have the smallest change and are always at the lower value, while the COD pollution volume in the western part is always at the higher value, and the overall change is more uniform. The amount of COD pollution in the three major sub-basins in the west, namely, the Zhuzhaoxin River, the Wanfu River, and the Dongyu River, was always at the downstream level from 2010 to 2020, and the results of the pollution treatment were not significant, and further treatment and restoration were needed in the future. The four sub-basins of the Zhushui River, the Baima River, the Beisha River, and the Chengguo River were always at the upper-middle level in the process of the change of the amount of COD pollution during the period of 2010–2020. The Xinxue River basin fluctuated considerably during this 11-year period, improving from the upper-middle level in 2010 to the lower-middle level in 2020.

**Fig 3:**
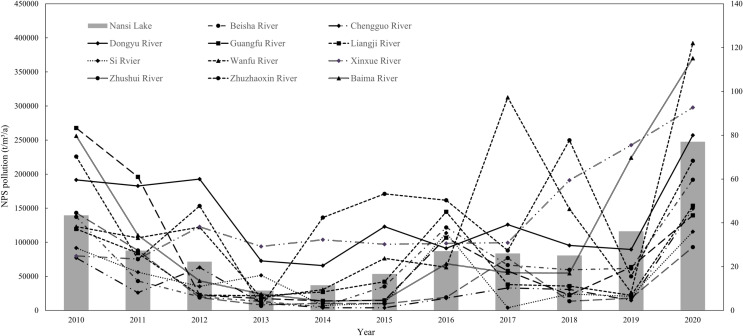
Temporal changes in total runoff NH_3_-N pollution in each sub-basin, 2010–2020.

The time-series course of NH_3_-N pollution loads in the NLB from 2010 to 2020 is shown in Fig 3 (Temporal changes in total runoff NH_3_-N pollution in each sub-basin, 2010–2020). The overall change in NH_3_-N pollutant discharges was uneven over the study period. For each sub-basin, the Guangfu River Basin shifted from a downstream level in 2010 to an upstream level in 2018, and maintained to an upper-middle level in 2020. Improvement was more pronounced in the Zhushui River Basin, which gradually improved from the downstream level in 2010 and returned to the upstream level by 2020. The overall change in the Xinxue River Basin is not promising, being at the upstream level in 2010 and changing to the midstream level by 2012–2014. Although it recovered in 2016, it gradually receded to the downstream level after 2018, and further management of NH_3_-N pollutants in the basin is needed.

### 3.3. Spatial variation of total NPS pollution in the Nansi Lake sub-basin

The spatial changes in the number of COD NPS pollution in the basin of the Nansi Lake from 2010 to 2020 are shown in Table 5. Overall, the NPS pollution in the central part of the basin is relatively good, and the spatial distribution of pollution is generally characterized as ‘low in the middle, high on both sides, and more concentrated’. The NPS pollution in the Zhushui River, Baima River, and Beisha River in the central part of the basin is always at a low value, while the COD pollution in the Zhuzhaoxin River, Wanfu River, and Dongyu River in the western part of the basin is always at the maximum value; the three basins with the largest COD NPS pollution in 2010 are Dongyu River, Wanfu River, and Zhuzhaoxin River in the order of magnitude. In 2020, the largest number of COD NPS pollution in NLB is still Dongyu River, which shows a significant increase compared with the amount of pollution in 2010. However, the basin with the smallest amount of COD NPS pollution in 2020 will still be the Zhushui River, the Baima River, and the Beisha River, and the amount of pollution will not fluctuate too much compared with that in 2010.

**Table 5:** Spatial variation in total runoff COD and NH_3_-N pollution by sub-basin.

Basin control unit	NPS COD pollution			NPS NH_3_-N pollution		
	2010	2015	2020	2010	2015	2020
Baima River	3390241.57	500245.39	3214485.21	79690.97	4662.64	115125.12
Beisha River	3001108.89	663579.59	4157904.82	137308.39	35088.30	191669.76
Chengguo River	1698922.51	431232.56	6200647.34	77136.95	3799.60	149261.23
Dongyu River	26294560.91	5376567.34	41274035.74	191442.92	122970.48	256984.81
Guangfu River	13863814.00	768786.23	7205661.35	267613.17	14666.19	139600.97
Liangji River	7308127.96	1844075.41	9809169.23	119615.14	42070.77	153641.17
Si River	8577944.53	1253949.17	11055870.01	91570.10	10499.30	115678.35
Wanfu River	14203462.48	2418552.14	14116911.26	122892.72	76462.04	392245.91
Xinxue River	3450505.51	2862605.52	33918028.73	79808.10	97099.20	897706.66
Zhushui River	3301641.40	417280.02	3060958.97	143188.85	9878.36	92868.60
Zhuzhaoxin River	9318503.39	10535105.37	35175967.05	225815.73	171097.01	219509.44

In the east-central part of the basin, the amount of NH_3_-N pollution was generally good, and the basin with the most obvious change in the amount of NH_3_-N pollution between 2010 and 2020 was the Xinxue River, which showed an increasing trend in the amount of NH_3_-N pollution. The three sub-basins with the largest amount of NH_3_-N NPS pollution in the NLB in 2010 were the Zhuzhaoxin River, the Dongyue River, and the Guangfu River in that order, while the three sub-basins with the smallest amount were the Xinxue River, the Chengguo River, and the Baima River in that order. The three sub-basins with the highest amount of NH_3_-N NPS pollution in the NLB in 2010 were Xinxue River, Zhuzhaoxin River and Dongyu River, while the three sub-basins with the lowest amount of pollution were Xinxue River, Chengguo River and Baima River, in order.

From the sensitivity test of each key factor of each subsystem above, the rate of change of population, the rate of change of GDP, the rate of rural sewage treatment, the proportion of the area of soil application of fertilizer, the rate of change of the number of livestock and poultry farming, and the rate of treatment of livestock and poultry farming on a large scale are the key and sensitive factors.

### 3.4. Analysis of factors affecting NPS pollution in the NLB

To further investigate the correlation between socio-economic factors and pollutants, this study analyzed the correlation between two pollutants, NH_3_-N and COD, and the influencing factors from 2010 to 2020 using the Pearson’s coefficient model of SPSS. Overall, both NH_3_-N and COD pollutants were significantly correlated with PAA, FAA, YAP and YTP from 2010 to 2020, with significant positive correlations with PAA and FAA. This result indicates that as the amount of annual fertilizer and annual pesticide increases, the pollutant content also increases, and the excessive use of fertilizer and pesticide can cause some NPS pollution. Meanwhile, the 2 pollutants were almost significantly negatively correlated with PTR, indicating that the pollutant content tends to decrease with the growth of the proportion of tertiary industry. The correlation coefficients between the characteristic factors such as GDP, YAP and PD were not consistent due to the different degrees of NPS pollution of NH_3_-N and COD pollutants in various site types. For COD pollutants, the main positive influencing factors of COD pollutants are YTP, FAA, RECA, LSI, and DA, with DA having the largest correlation coefficient of 0.92. The main negative influencing factors are NAPR, and SOMC. The main positive impact factors of NH_3_-N pollutants were YTP, YAP, FAA, RECA, LSI, and DA, and the main negative impact factors were PPR (−0.340) and SOMC (−0.449).

**Fig 4:**
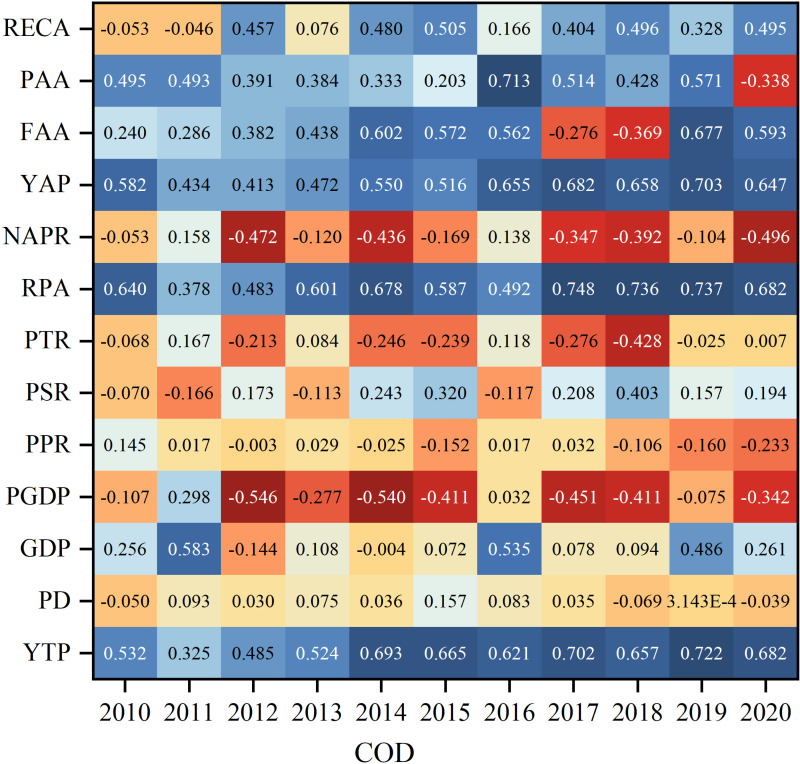
Correlation coefficients between socio-economic factors and COD pollutants.

COD is an important indicator for responding to organic matter in water bodies, and most of the influencing factors showed significant positive correlations with COD. Specifically, COD was consistently positively correlated with YTP and RPA, indicating that COD was more associated with demographic factors, showing a significant positive correlation. In 2010, the correlation coefficients between COD pollutants and RPA, YAP, PAA, and YTP characteristic factors were all positive, and all exceeded 0.5. The correlations of the factors that showed negative correlations in 2010 were all less than 0.11. By 2015, PSR and RECA were the most significantly changed influencing factors, which changed from a lower negative correlation to a positive correlation. In addition, the changes of PGDP, PSR and FAA gradually changed from a low correlation to a high correlation. The correlation between the influencing factors and COD pollutants changed less during the period of 2015–2020, which may be due to the fact that the socio-economic situation gradually changed from a period of rapid development to a period of stabilization, and the correlation between the factors has formed a more stable relationship (Fig 4 Correlation coefficients between socio-economic factors and COD pollutants).

**Fig 5:**
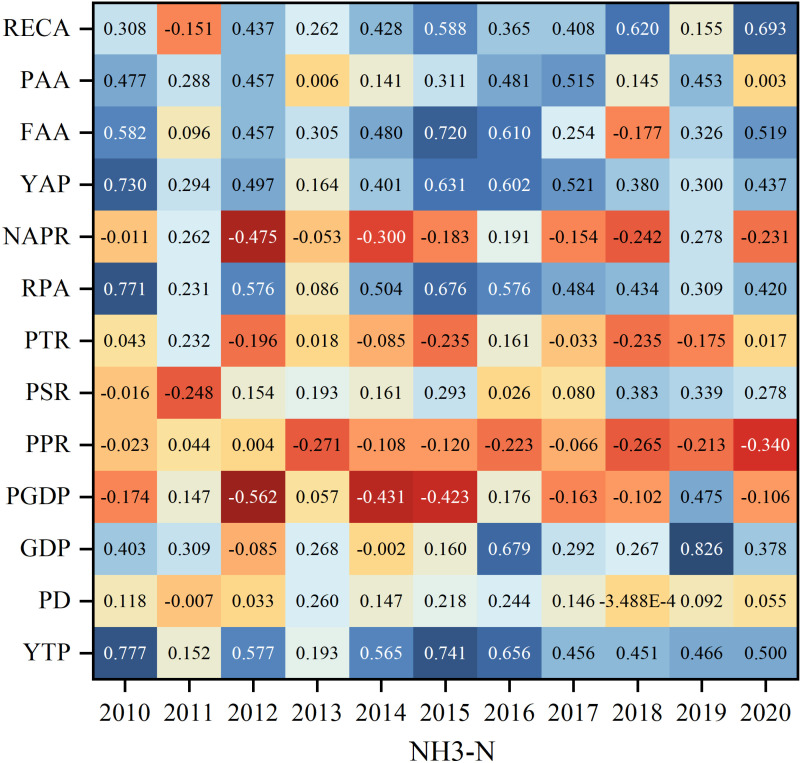
Correlation coefficients between socio-economic factors and NH_3_-N pollutants.

During the period 2010–2020, NH_3_-N pollutants are positively correlated with YTP and the correlation coefficients are large, indicating that the total population is an important factor affecting the emission of NH_3_-N pollutants, in addition to this, the positive correlation between NH_3_-N pollutants and RECA is more obvious, indicating that as the total amount of electricity consumption in the rural areas increases, the production of NH_3_-N pollutants is also higher, and the electricity consumption in the rural areas has a negative impact on NH_3_-N has an adverse effect, while the correlation coefficients of NH_3_-N pollutants with PPR and NAPR are mostly negative, indicating that the increase in the proportion of non-agricultural population and the development of primary industry are favorable to NH_3_-N emissions, which may be that the more developed the plantation and forestry industry is, the stronger the fixation of ammonia nitrogen is, which makes the pollutants in the air less. In 2010, the positive correlation coefficient between NH_3_-N and YTP and RPA characteristic factors was the largest, reaching more than 0.7. In 2015, PGDP was significantly negatively correlated with NH_3_-N (−0.4230), indicating that as GDP per capita increases, it leads to a decrease in NH_3_-N emissions. In 2020, there was a negative correlation between NH_3_-N pollutants and three characterization factors, NAPR, PPR and PGDP, and a positive correlation with all other characterization factors (Fig 5 Correlation coefficients between socio-economic factors and NH_3_-N pollutants).

**Fig 6:**
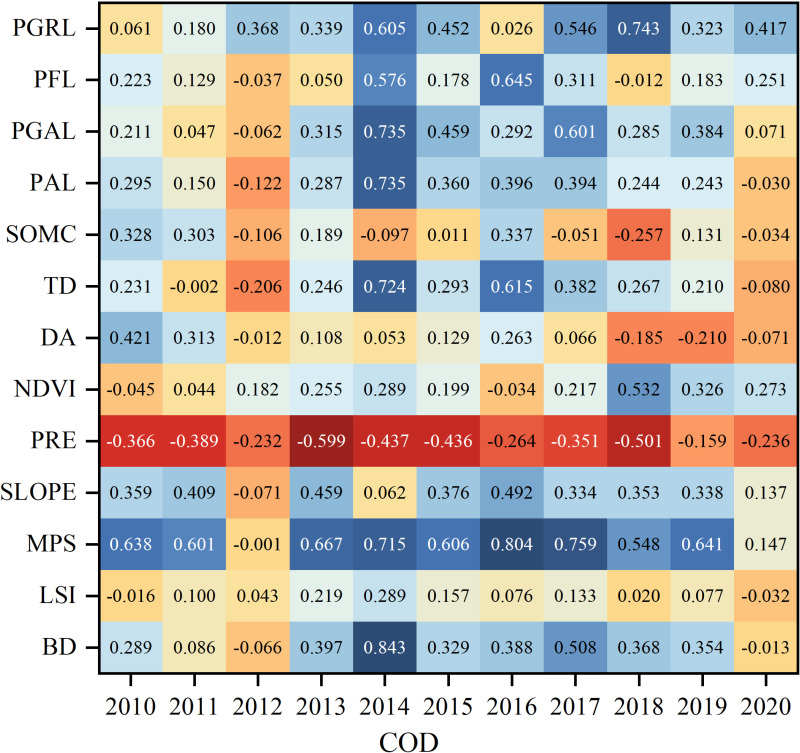
Correlation coefficients between natural factors and COD pollutants.

Most of the natural factors were positively correlated with COD, and only 5 out of 13 natural factors were negatively correlated with it. LSI had the most obvious positive correlation, with a Pearson coefficient as high as 0.5747, which is due to the fact that when the complexity of the local landscape increases, it increases the possibility of contact between the landforms and the water body and the possibility of pollutants flowing into the water body. PRE and PFL also have the highest positive correlation with COD, and their Pearson coefficients exceeded 0.5. PRE and PFL are also the natural factors with the highest positive correlation with COD, with Pearson’s coefficients exceeding 0.5, as the scouring effect of precipitation and runoff transport lead to increased pollutant levels in water bodies (Fig 6 Correlation coefficients between natural factors and COD pollutants).

**Fig 7:**
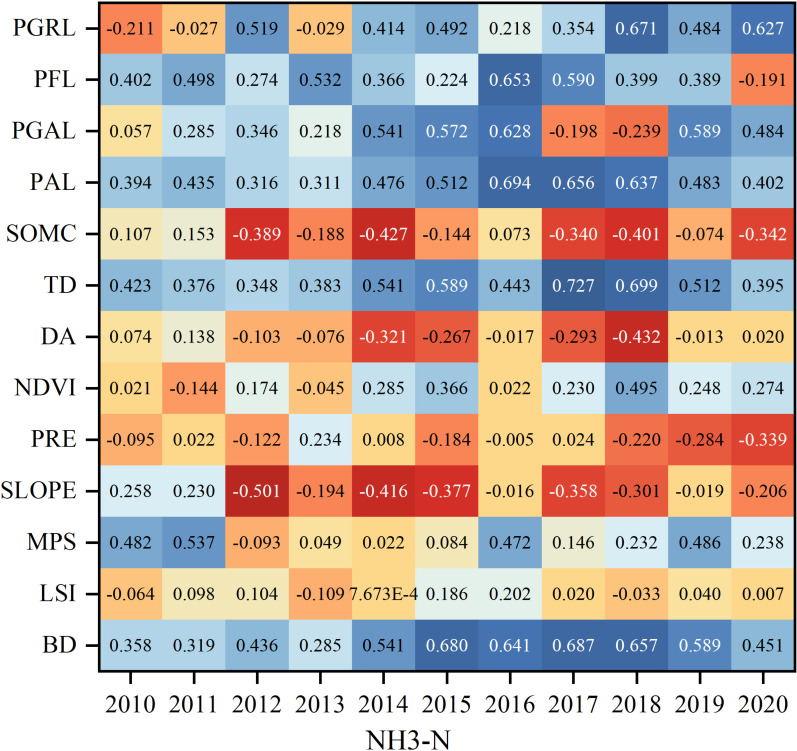
Correlation coefficients between natural factors and NH_3_-N pollutants.

Agricultural NPS pollutants, particularly NH_3_-N, show a mostly positive correlation with natural factors. Among these, NH_3_-N is significantly positively correlated with the natural factors LSI and DA, with correlation coefficients exceeding 0.5, reaching 0.7050 and 0.6630, respectively. Pollutant emissions are also closely related to DA, pollutant transport distance, and the retention time within the basin. The larger the area, the greater the pollutant retention and degradation, which in turn leads to increased NH_3_-N pollutant production. Furthermore, TD is another natural factor affecting pollutant discharge. A higher density of trenches results in organic matter, NH_3_-N, and other pollutants from farmland drainage being directly transported into nearby water bodies, leading to a higher subsequent generation of pollutants. NH_3_-N pollutants are significantly negatively correlated with natural factors such as SOMC and PGRL. Specifically, higher organic matter content in the soil is associated with lower NH_3_-N pollution levels. Additionally, as polar organic polymers increase, pollutant degradation becomes stronger, leading to reduced pollutant production. Moreover, a higher PGRL is associated with lower NH_3_-N concentrations, indicating that grasslands have a stronger pollutant degradation effect (Fig 7 Correlation coefficients between natural factors and NH_3_-N pollutants).

### 3.5. Analysis of regression modelling

Using two regression models (Equations 6 and 7), eight basin characteristics were selected as independent variables, while COD and NH_3_-N NPS pollution amounts from 2010 to 2020 were chosen as the dependent variables. Prediction models were established, and multiple linear regression analyses were conducted, resulting in the regression equations shown in Table 6. After testing for significance, the individual regression equations passed the significance level test (P < 0.05).

**Table 6:** Regression equations for NPS pollutants.

Name	Equation	R^2^
NH_3_-N (1a)	${\text{L}}_{\text{a}}=1.070{\text{X}}_{1}-0.835{\text{X}}_{8}-0.138{\text{X}}_{9}+0.199{\text{X}}_{10}+0.101{\text{X}}_{13}+0.342{\text{X}}_{15}+0.454{\text{X}}_{20}+0.058{\text{X}}_{21}+0.141{\text{X}}_{22}$	0.811
COD (1b)	${\text{L}}_{\text{a}}=0.572{\text{X}}_{1}+1.139{\text{X}}_{8}-0.074{\text{X}}_{9}-1.024{\text{X}}_{10}+0.038{\text{X}}_{13}-0.309{\text{X}}_{15}+1.400{\text{X}}_{20}-0.209{\text{X}}_{21}+0.229{\text{X}}_{22}$	0.698
NH_3_-N (2a)	$\text{log}({\text{L}}_{\text{b}})=0.023\text{log}\left({\text{X}}_{1}\right)-0.138\text{log}({\text{X}}_{8})+0.121\text{log}({\text{X}}_{10})+0.198\text{log}\left({\text{X}}_{13}\right)-1.215\text{log}\left({\text{X}}_{15}\right)-0.831\text{log}\left({\text{X}}_{20}\right)-0.616\text{log}({\text{X}}_{21})+0.457\text{log}({\text{X}}_{22}$	0.763
COD (2b)	$\text{log}({\text{L}}_{\text{b}})=0.275\text{log}\left({\text{X}}_{1}\right)-0.074\text{log}({\text{X}}_{8})-0.136\text{log}({\text{X}}_{10})+0.197\text{log}\left({\text{X}}_{13}\right)-1.263\text{log}\left({\text{X}}_{15}\right)-0.953\text{log}\left({\text{X}}_{20}\right)-0.445\text{log}({\text{X}}_{21})+0.513\text{log}\left({\text{X}}_{22}\right)$	0.821

Models 1a and 1b were developed based on NH_3_-N and COD NPS pollution amounts in Equation 6, while models 2a and 2b were based on NH_3_-N and COD NPS pollution amounts in Equation 7 (Table 6).

## 4. Discussion

### 4.1. Validation of model results

The results of NPS pollution division were tested by local government statistics and related studies, and the average of the total COD and NH_3_-N pollution and NPS pollution share of the 11 control units were taken as the total pollution and NPS pollution share of the NLB. In terms of individual control units, the average NPS pollution amount of NH_3_-N in Dongyu River was 135.19 t, with Congmin [41] 70.14 t, with an error of 48.12%, the NH_3_-N pollution amount of Zhuzhaoxin River was 139.74 t, with Xiao [42] 86.81 t, with an error of 37.88%, the average NPS pollution amount of NH_3_-N in Xinxue River was 245.61 t, with Zhang et al. [43] 337.34 t, the error is 27.19%.

NLB simulation 2010–2020 average COD amount of 52668.24 t, with Wang et al. [44] 2005–2012 average NPS pollution amount of 78215.69 t, the error is 32.66%, with Ni et al. [42] 2007 COD NPS pollution reference 53442.47 t, the relative error is 1.45%, with Zhang et al. [43] 46803.15 t with a relative error of 11.14%, and with Wang [44] 64092.9 t with a relative error of 17.83%.

The simulated NPS pollution of COD and NH_3_-N in Nansi Lake accounted for 78.77% and 70.44%, respectively, with Congmin [41] 67.90% and 60.29% observations with errors of 10.87% and 10.15%, respectively. The proportion of NPS pollution in Nansi Lake is 65.05% and the proportion of NPS pollution discharge from living agriculture in Corwin [45] is 67.22%, with an error of 2.17%; therefore, the reliability of the simulation results meets the requirements of the analysis.

### 4.2. Validation of multiple regression equations

The regression equations were tested for prediction by simulating the amount of NPS pollution in Dongyu River, Chengguo River, Baima River and Beisha River from 2010 to 2020. As shown in Tables 7 and 8, in general, RE values between 0–0.3, R^2^ values above 0.5, and ENS values between 0–1 indicate that the fitness test of the model was passed [43]. The test results showed that models 1a and 2a simulated NH_3_-N NPS pollution in the Dongyu River with a large RE, and model 1a simulated the Baima River Basin with an RE also greater than 0.3 and an R^2^ less than 0.5, which is a large error, but in testing the rest of the control units. Models 1a and 2a had R^2^ values between 0.62–0.89 and 0.51–0.67, respectively, and ENS was also between 0 and 1, simulating well. Except for model 1b which simulated COD NPS pollution in Dongyu River with RE greater than 0.3 and R^2^ value of 0.48. In testing the rest of the control units, RE, R^2^, and ENS passed the suitability test, which indicated that models 1a, 2a, and 2b were better suited to simulate the amount of NPS pollution in the NLB.

**Table 7:** Regression model 1a and 2a tests.

Basin	1a			2a		
	RE	R^2^	ENS	RE	R^2^	ENS
Dongyu River	−0.39	0.74	0.13	−0.34	0.62	0.89
Chengguo River	0.15	0.62	0.56	−0.26	0.51	0.22
Baima River	−0.47	0.81	0.06	−0.21	0.38	0.31
Beisha River	0.13	0.89	0.95	0.33	0.67	0.20

**Table 8:** Regression model 1b and 2b tests.

Basin	1b			2b		
	RE	R^2^	ENS	RE	R^2^	ENS
Dongyu River	−0.75	0.48	0.05	−0.11	0.64	0.86
Chengguo River	−0.17	0.62	0.27	−0.01	0.75	0.85
Baima River	−0.02	0.50	0.04	0.02	0.55	0.72
Beisha River	0.25	0.73	0.69	0.01	0.63	0.87

### 4.3. NPS pollution sources

Through the analysis of the NH_3_-N emission accounting process, it was found that the average annual total NH_3_-N discharged into the water environment from pollution sources in the NLB from 2010 to 2020 was 1,468.26 t, while the average annual total COD discharge was 735,519.79 t. Therefore, COD is the primary pollutant in the NLB. Table 6 illustrate the spatial distribution of COD and NH_3_-N pollution across the sub-basins of Nansi Lake. The spatial variation of NPS pollutants across regions was significant, with the highest pollution levels found in the Dongyu River Basin, where land use is predominantly arable. Most of the COD and NH_3_-N entered the water through sediment adsorption, aligning with the results of previous studies [46,47]. The contribution rates of NH_3_-N and COD pollution from NPS sources flowing into rivers were calculated for different areas, showing that Heze had the highest total NH_3_-N pollution, while Tai’an had the lowest, consistent with Zhang et al. [32]. In the northeastern part of the NLB, where land use is dominated by forests and grasslands, vegetation effectively retains water, resulting in lower pollutant flow [48]. Excess fertilizers, rural households, and dispersed livestock farms make up a significant portion of the basin [49].

### 4.4. NPS pollution influences and basin planning

Exploring the similarities between anthropogenic and intrinsic basin factors (such as soil type, climate, topography, and hydrology) is critical. Zoning based on similar characteristics can be used to assess pollutant loss [50,51], evaluate NPS pollution risks [34,52], and implement effective management practices for NPS pollution control [53,54]. The 11 sub-basins show high levels of NH_3_-N and COD pollution with notable seasonality, consistent with previous findings [27]. Pearson’s correlation analysis revealed a significant correlation between NH_3_-N and COD pollution and the PAL. NH_3_-N displayed a positive correlation in all studied years, while COD exhibited a negative correlation in 2012 and 2020 but was positively correlated in other years, differing from earlier studies [32]. This suggests that NH_3_-N and COD pollution is higher in cultivation-dominated basins, a result comparable to findings reported in the literature [55,56]. The correlation between NH_3_-N and COD pollution and the PFL was also significant, with a positive correlation in most years. Studies have shown that forests play an important role in reducing nitrogen loads [57]. Prolonged flooding processes, due to the hydrological buffering effects of forests, can reduce NH_3_-N export by slowing down runoff and extending the time for pollutant transport to streams. Seitzinger et al. [58] found that 37% to 76% of NH_3_-N input into streams is removed during transport, which contrasts with our findings. Moreover, the correlations between COD and NH_3_-N loads with factors such as YTP, RPA, LSI, and DA were significantly linear, confirming that the primary source of pollution stems from human activities, a conclusion that differs from previous studies [59–61].

### 4.5. Study limitations and future directions

For the management of NPS pollution in the NLB, the L-THIA model was used to calculate the total amount of NPS pollution and to analyze the influencing factors of NPS pollutants, and finally a linear equation was established to fit a multiple regression model, with a view to balancing the good interaction between agricultural production and the ecological environment, and to laying a good theoretical foundation for the sustainable development of the basin. However, the following limitations should be addressed in order to better provide new avenues for future research. The period of high NH_3_-N loss mainly occurs in the rainy season (March ~  June) and the period of high NH_3_-N enrichment mainly occurs in the dry season (January ~  February). This temporal difference in monthly variation shows a clear transport characteristic of NPS pollution, as pollutant transport in NPS occurs mainly in the rainy season [47], our analyses are mainly year-based and the effects of monthly variations in the rainy versus dry seasons should be considered later. In addition the effects of heavy rainfall processes on COD and NH_3_-N [50] should also be considered in future studies.

## 5. Conclusions

Based on the runoff partitioning method, a data series of agricultural NPS pollution distribution in the basin was obtained to quantitatively assess the spatial and temporal distribution of agricultural NPS pollution in the basin and quantitatively analyze the natural and socio-economic influences affecting them. Finally, multiple regression analysis was conducted to construct regression equations and simulate the future agricultural NPS pollution. Agricultural pollution in the NLB takes 2014 as the dividing line, showing a trajectory change of decreasing and then increasing, and the magnitude of increasing and decreasing varies greatly. In terms of socio-economic influencing factors, both COD and NH_3_-N were significantly correlated with PAA, FAA, YAP and YTP. In terms of natural influences, LSI, DA and TD had higher positive correlation correlations with various pollutants, and slope and SOMC had higher negative correlations with pollutants. The study found that agricultural surface pollution in the NLB mainly originates from livestock and poultry farming, so it is necessary to carry out a comprehensive promotion of the cleanliness of farming production and the ecology of the industrial model. The relevant departments should guide farm households to carry out appropriate scale farming, promote the creation and enhancement of standardized farming demonstrations, and lead the green and sustainable development of the livestock industry. The government should also increase its support for the animal husbandry and veterinary industry, and improve the construction of the animal disease prevention and control system. In addition, there is a need to optimize and adjust the agricultural structure to achieve a balance between industrial returns and the ecological environment. As a large agricultural province in Shandong Province, rich in agricultural resources, the degree and direction of agricultural development in various regions of the province are also different, so the adjustment of the agricultural structure needs to be fully considered to achieve a combination of regional characteristics and the actual development of a reasonable allocation of resource elements, optimize the layout of productivity according to local conditions.

## Supporting information

S1 Data Data of this study. (XLSX)
